# Molecular-scale structures of the surface and hydration shell of bioinert mixed-charged self-assembled monolayers investigated by frequency modulation atomic force microscopy†

**DOI:** 10.1039/c8ra03569e

**Published:** 2018-07-10

**Authors:** Yuki Araki, Taito Sekine, Ryongsok Chang, Tomohiro Hayashi, Hiroshi Onishi

**Affiliations:** Department of Chemistry, School of Science, Kobe University Rokkodai, Nada Kobe Hyogo 657-8501 Japan yukiaraki@piezo.kuee.kyoto-u.ac.jp +81 75 383 2308 +81 75 383 2307; Department of Materials Science and Engineering, School of Materials and Chemical Technology, Tokyo Institute of Technology Nagatsutacho, Midoriku Yokohama Kanagawa 226-8502 Japan

## Abstract

We studied the surface structure and hydration structure of a bioinert mix-charged self-assembled monolayer (MC-SAM) comprised of sulfonic acid (SA)- and trimethylamine (TMA)-terminated thiols in liquid by frequency modulation atomic force microscopy (FM-AFM) at a molecular-scale. The TMA end groups showed a highly-ordered rectangular arrangement on a gold substrate in phosphate buffer saline (PBS). Highly structured water was observed at the interface of the MC-SAM and PBS, whereas a less structured hydration structure was observed on bioactive SAMs such as those with OH– and COO– terminal groups. Differences in surface and interface structures between the bioactive and bioinert SAMs suggest that the highly structured water at the bipolar MC-SAM surface works as a physical barrier to prevent adsorption or adhesion of protein and cells. Our results led to the idea that the hydration structure is an important factor in the determination of interactions between SAMs and biomolecules.

## Abstract 2

Water molecules above a bioinert mixed-charged self-assembled monolayer (MC-SAM) surface are highly structured compared to those of bioactive SAM surfaces.

## Graphical abstract

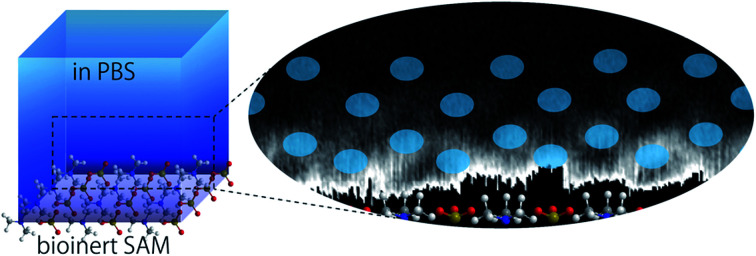

## Introduction

Self-assembled monolayers (SAMs) on solid substrates provide versatile platforms for fundamental and applied works ranging from self-assembly in 2D, to tribology, catalysis, biological assay, *etc.* In particular, SAMs have been employed as scaffolds for biomolecules and cells since the 1990s because of their flexibility in controlling the physicochemical properties of surface stability in water. However, understanding the interactions of SAMs with biomolecules and cells still involves difficulties, since the interfacial behavior of water and ions, as well as the packing structure of the molecules constituting the SAMs, are important factors that govern the interactions. Surface-sensitive analytical techniques such as sum frequency generation (SFG) and X-ray reflectivity spectroscopy revealed the local behavior of water molecules and ions in the vicinity of surfaces of solid materials.^1–4^ In the 2000s, the improvement of frequency modulation atomic force microscopy (FM-AFM) paved a way to investigate the spatial distribution of interfacial water molecules in three dimensions at sub-nanometer resolution. In particular, FM-AFM has revealed detailed structures of the interfacial water along with the packing structure of thiolate molecules of SAMs.^5–8^ Recently, interfacial water was found to play important roles in bioinertness (protein- and cell-resistance).^9^ Hayashi *et al.* reported that the interfacial water in the vicinity of SAMs of oligo (ethylene glycol)-terminated alkanethiols (OEG-SAMs) played a role as a physical barrier against the approach of proteins and cells toward the SAMs.^10,11^ Recently, the flexible movement of OEG groups in water has been visualized by FM-AFM and the relevance of this to their bioinertness was discussed.^12^ Collecting these previous results together, biointertness of the OEG-SAMs originates from the interfacial water and flexible terminal groups.

In this work, we focus on a bioinert mixed-charged SAM (MC-SAM) consisting of two thiols whose terminal groups are sulfonic acid (SO_3_^−^: SA) and trimethylamine (N^+^(CH_3_)_3_: TMA) groups. Chen *et al.* already reported that TMA and SA molecules are organized into a MC-SAM *via* strong charge–charge interactions in water, and that the SAM had a rigid and highly-crystalline structure as was revealed by AFM in a contact mode.^13^ Therefore, flexible movements of the terminal groups cannot be an origin of its bioinertness. Hence, we conducted molecular-scale observations of the MC-SAM surface by using a non-contact FM-AFM technique to visualize the real surface structure in liquid. Furthermore, the structure of the interfacial water in the vicinity of the SAM was analysed and we discuss the mechanism underlying its bioinertness.

## Experimental

### Preparation of the MC-SAM

Au (111) substrates were prepared by vacuum deposition of gold onto freshly cleaved mica (10 × 10 × 0.3 mm, S & J TRADING INC.) at 620 K under a vacuum pressure of 10^−5^–10^−6^ Pa. The substrates were then annealed at 620 K in a vacuum chamber for 2 h. Next, MC-SAMs were prepared on the Au (111) substrates. Chemical structures of the alkanethiol molecules used are shown in Table 1. All molecules were purchased from ProChimia Surfaces (Poland). MC-SAMs were formed by immersion of the Au (111) substrates into freshly prepared 1% ammonia water containing the SA and TMA molecules, both at a concentration of 1 mM. After immersion for 24 h, the substrates were rinsed with pure ethanol to remove excess alkanethiol molecules from the surfaces of the SAMs. The formation of MC-SAMs with TMA : SA = 1 : 1 was confirmed by X-ray photoelectron spectroscopy (XPS) measurements (Theta Probe, Thermo-Fisher Scientific Inc., MA, USA) as shown in the ESI (Fig. S1 and S2†).

**Table 1:** List of alkanethiol molecules used in this study

Abbreviation	Chemical structure of the alkanethiol molecules
SA	HS–(CH_2_)_11_–SO_3_^−^
TMA	HS–(CH_2_)_11_–N^+^(CH_3_)_3_

### FM-AFM

Frequency modulation mode atomic force microscopy (FM-AFM) is a technique used to observe surface and interface structures at the atomic/molecular scale. The frequency shift (Δ*f*) of the cantilever oscillation is detected under lower noise and higher sensitivity in the vicinity of a sample surface.^14^ Inorganic^15,16^ and organic^17,18^ substrate surfaces at the atomic/molecular scale have been visualized by this method in liquid. For the last decade, observations of molecular-scale structures of solid–liquid interfaces have also been achieved by Δ*f* mapping^19^ as mentioned in the next section. We used FM-AFM equipment compatible with SPM-8100FM (Shimadzu, Japan). We employed silicon cantilevers with backsides coated with gold (PPP-NCHAuD, Nanosensors, Switzerland). The eigenfrequency of the cantilevers, the quality factor, and the spring constant were 166 kHz, around 8, and 40 N m^−1^ in aqueous solution, respectively. The FM-AFM equipment was located in an incubator (CN-40A, Mitsubishi Electric Engineering, Japan) to maintain the temperature at 22 °C.

### Δ*f* mapping

Hydration structures at the SAM–liquid interfaces were observed by Δ*f* mapping.^19^ The distance dependency of Δ*f* of the cantilever oscillation (Δ*f*–distance curve) was recorded while the cantilever tip approached and retracted from the sample surface and the bulk liquid with a peak-to-peak amplitude of 0.3–0.4 nm. The Δ*f* was positively and negatively shifted by the repulsive and attractive forces to the tip, respectively. The tip started to retract from the sample surface when the Δ*f* reached the threshold value (>0 Hz). A two-dimensional cross-sectional image of the solid–liquid interface consisted of 256 Δ*f*–distance curves measured in intervals of 0.1 nm to the width direction. The scanning area was 2.5 nm × 5.0 nm (height × width).

### Procedure

The MC-SAM substrate was glued to an open fluid cell with a diameter of 15 mm with an epoxy adhesive. The MC-SAM surface was observed in a meniscus of 200 μL phosphate buffer saline (PBS) (Sigma-Aldrich) after the surface was rinsed with pure ethanol just before the experiment. All experiments were done at room temperature. The surface topography and interfacial images were observed with peak-to-peak oscillation amplitudes of 1–2 nm and 0.3–0.4 nm, respectively. The WSxM software package (Nanotech Electronica)^20^ was used for image rendering and data processing.

## Results and discussion

**Fig. 1:**
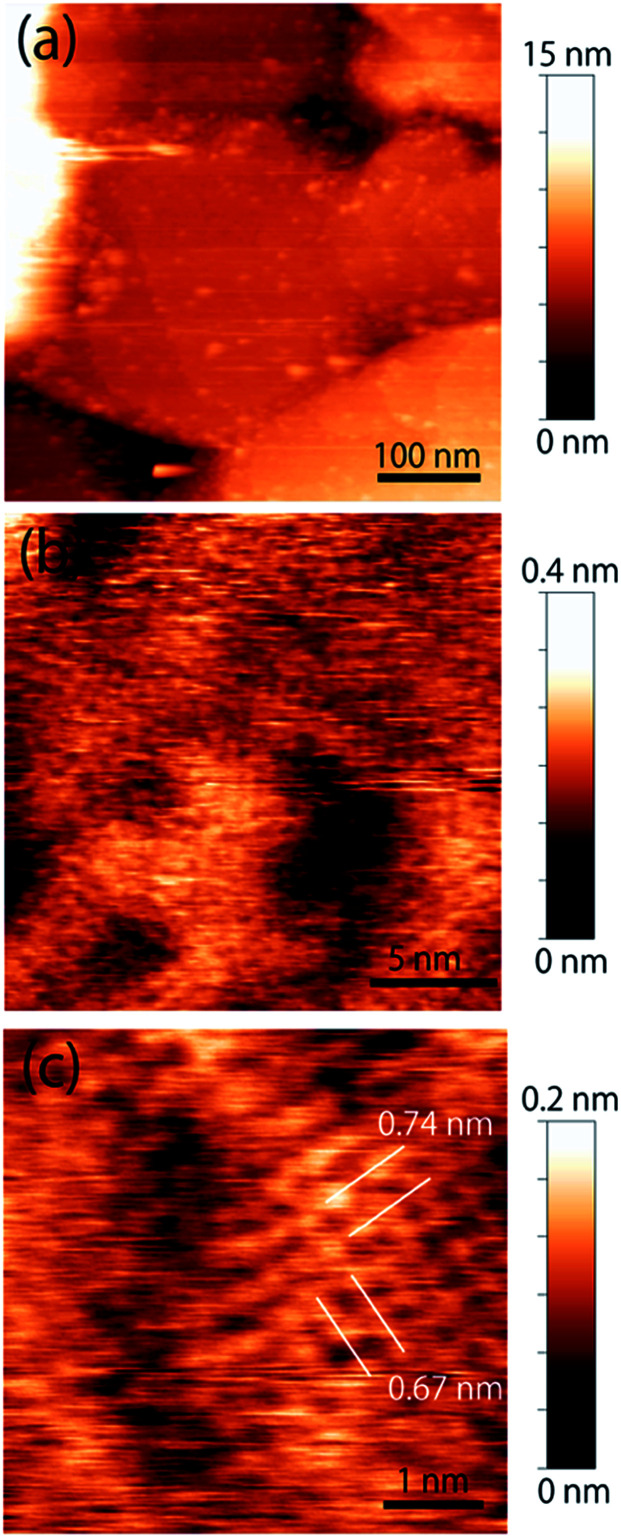
Topographic images of the MC-SAM surface. (a) MC-SAM islands on the gold surface obtained with a peak-to-peak amplitude of 2 nm. (b) Magnified image of the MC-SAM surfaces. (c) Molecular-scale image of the terraces. Images (b) and (c) were obtained with a peak-to-peak amplitude of 1 nm and a threshold Δ*f* of 1.3 kHz.

**Fig. 2:**
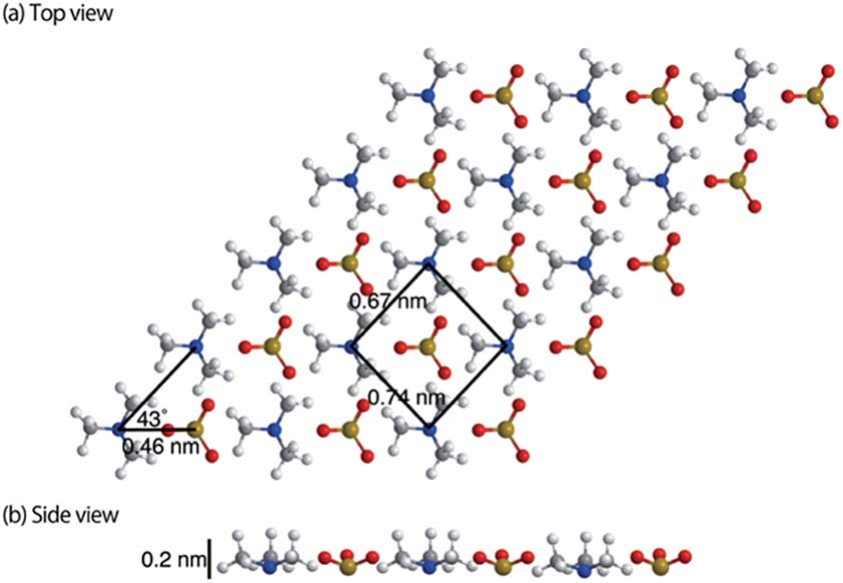
Schematic image of the MC-SAM surface. The foreground and the interior molecules are TMA and SA, respectively. It should be noted that the structure was illustrated only by considering the sizes of the unit cell and the molecules and that their geometries are not optimized by energy calculations.

The topographic image of the MC-SAM on the gold surface is shown in Fig. 1(a). We confirmed a periodic structure in the magnified images in Fig. 1(b and c). Our observations revealed a rectangular unit lattice with a size of 0.74 × 0.67 nm shown in Fig. 1(c). By considering that alternate positioning of TMA and SA packed in the SAM provides the lowest free energy of the SAM, we proposed a model as shown in Fig. 2(a). The molecular size of the TMA moiety is much larger than that of SA, as shown in Fig. 2(b), and the height difference between bright and dark areas in the unit cell is in good agreement with the differences in the sizes of the moieties (about 0.2 nm). Therefore, we concluded that bright spots correspond to the positions of TMA molecules. Collecting these findings together, we proposed the model shown in Fig. 2. Although we observed the lattice structure of the MC-SAM, there is a discrepancy between our results and the structure reported by Chen *et al.* regarding the shape of the unit cell.^13^ AFM images obtained with a contact mode in air showed a parallelogram shape, whereas our result showed a rectangular shape. Note that we confirmed that the scanning direction in a vertical axis (slow scan axis) did not affect the images, indicating that our images were free from the effect of thermal drift as shown in Fig S3.† We assume that there are two possible reasons for the origin of the discrepancy. One is the difference in the measuring mode. For imaging in the contact mode, a considerable force is applied to the sample from the probe, inducing distortion of the sample. On the other hand, the non-contact FM mode (our work) minimizes the probe–surface interactions. Therefore, we believe that this is the reason for the discrepancy. The other reason for the discrepancy is the difference between our and their measuring environment. In contrast with the measurements under ambient conditions, the redistribution of water and ions in the interfacial region occurs in water, which will be discussed in the next section.

**Fig. 3:**
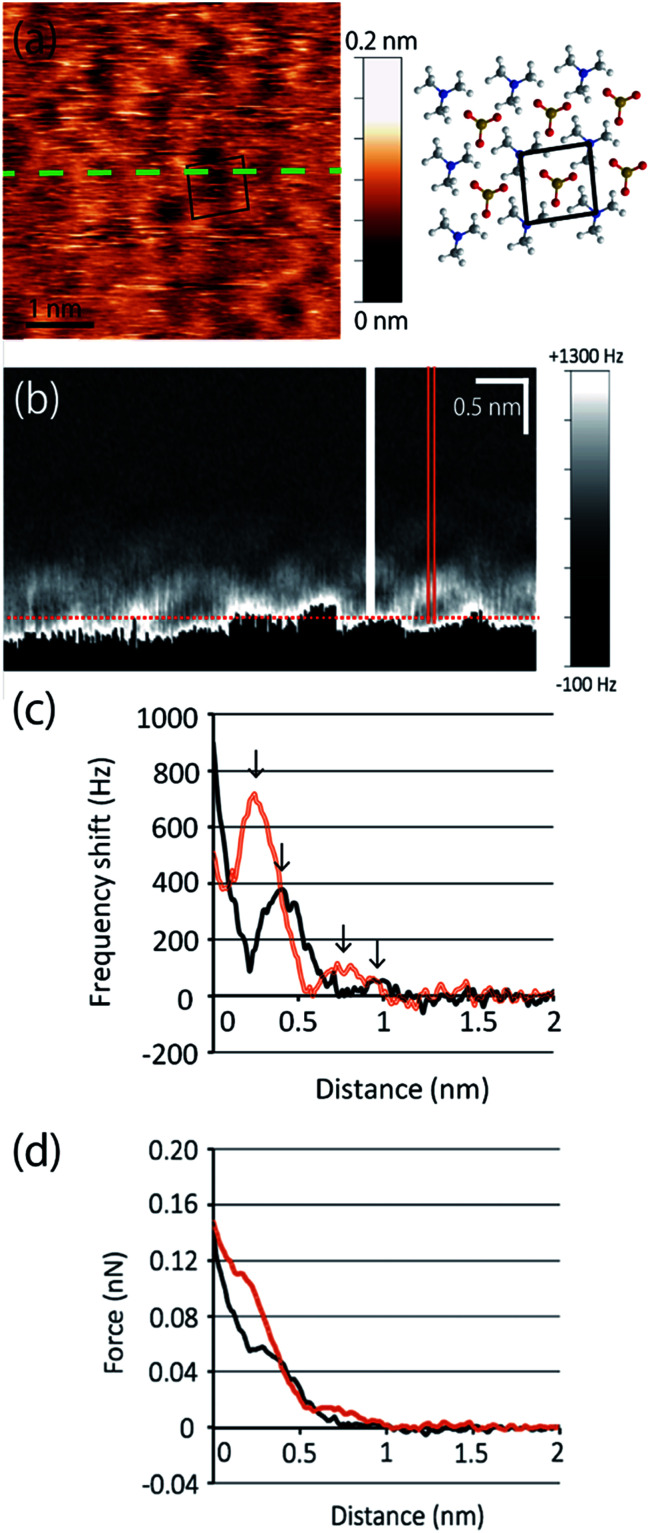
(a) Topographic image of the MC-SAM. The black box indicates the unit cell. (b) Interfacial image at the MC-SAM and PBS interface along the dashed line in (a). Location of distance = 0 was indicated by a dotted line. (c) Δ*f*–distance curves. Arrows indicate the repulsive peaks. (d) Force–distance curves. The black and orange line curves in (c) and (d) were obtained from the filled line and the double line in (b), respectively.

Next, we extend our discussion to the hydration structure of the MC-SAM. The Δ*f* map of the solid–liquid interface, Fig. 3(a), and its cross-sectional image, Fig. 3(b), are shown. The cross-sectional image of the interface of the MC-SAM and PBS, Fig. 3(b), was observed along the middle line of Fig. 3(a). The distance of these protrusions (0.6 nm) is consistent with the intermolecular distance between TMA molecules. We observed the rectangular structures at a peak-to-peak amplitude. These brighter areas represent the positive Δ*f* that indicates repulsion from the water molecules in the vicinity of the MC-SAM surface. The Δ*f*–distance curves measured above the protrusions (TMA) and the gaps (SA) of the MC-SAM surface showed two peaks, respectively (Fig. 3(c)). The distance between the peaks (indicated by arrows in Fig. 3(c)) was about 0.2–0.3 nm, which is close to the size of a single water molecule. Combining these results and our previous findings, we can conclude that the pattern of the bright areas in Δ*f* map represents the hydration structure of the MC-SAM. In addition, we compared the oscillation peaks of the Δ*f* curve with the force curves (Fig. 3(d)). The Δ*f* was converted to interaction force according to the Sader and Jarvis equation.^22^ The positions of the repulsive peaks in the Δ*f* and force curves corresponded, although the peaks of the third and fourth layers were weak in the force curves. This finding supports that the oscillation peaks of Δ*f* curve indicate the existence of hydration layers. Based on the above discussion, our Δ*f* map suggests that the formation of a hydrogen bonding network of the interfacial water is driven by the SAM. This structure is also found for other hydrophilic surfaces, *e.g.*, mica, calcite, and other SAMs.^5,6,19,21^

The hydration structure of the MC-SAM is different from that of the SAMs of hydroxyl-, carboxyl- and methyl-terminated alkanethiols (OH–SAM, COOH–SAM and CH_3_–SAM respectively). The prominent difference is that the structures of the second and third hydration layers of the MC-SAM showed clearer structure in the lateral direction than those of the OH– and COOH–SAMs in terms of Δ*f*.^5^ In addition, the repulsive force by the first hydration layer was two orders of magnitude larger than that of the CH_3_–SAM surface which used the same type of Si-cantilever as our system.^6^ Although the individual differences of tip shape should be considered to discuss the interaction force quantitatively, the differences in the orders of magnitude must not be only due to the tip-shape differences but also due to the stronger interactions of the surface molecules and the closest water molecules. This indicates that water molecules in the vicinity of the MC-SAM were more tightly immobilized and highly structured in the interfacial region with a thickness of 1 nm.

Finally, we discuss the relevance of the hydration structure to the bioinertness (protein- or cell-resistance) of SAMs. In contrast with the MC–SAM, OH– and COOH–SAMs are protein-adsorbing and cell-adhering surfaces.^10^ Recently, it was found that interfacial water molecules in the vicinity of various bioinert SAMs act as a physical barrier to prevent adsorption and adhesion of proteins and cells, and this was confirmed by both theoretical and experimental approaches.^10,11,23–26^ Our FM-AFM results also revealed mechanically stable water layers in the vicinity of the bioinert MC-SAM in physiological conditions at a sub-nanometer scale.

## Conclusions

We successfully observed a molecular-scale structure of the MC-SAM surface in water by non-contact FM-AFM. We found that TMA and SA molecules are assembled in a rectangular arrangement in the MC-SAM in contrast with the hexagonal packing patterns found for other SAMs of alkanethiols. Furthermore, our measurements revealed the hydration structure at the interface between the MC-SAM and water at a sub-molecular scale. The important finding here is that the mechanical stability of the hydration shell in the vicinity of the MC-SAM is much higher than that of protein-adsorbing OH– and COOH–terminated SAMs. Together with previous experimental and computational works, we concluded that the hydration shell of the MC-SAM is the origin of the protein and cell resistance. In future, we expect that non-contact FM-AFM may pave a way to reveal the underlying mechanisms of wetting, biocompatibility and other complex interfacial molecular processes at various biointerfaces both qualitatively and quantitatively.

## Conflicts of interest

There are no conflicts to declare.

## Supplementary Material

RA-008-C8RA03569E-s001
