# Supplementary material for: Molecular-scale structures of the surface and hydration shell of bioinert mixed-charged self-assembled monolayers investigated by frequency modulation atomic force microscopy
Source: RSC Adv. 2018 Jul 10;8(43):24660–4. doi: 10.1039/c8ra03569e (PMC9082155; doi:10.1039/c8ra03569e)
Supplement: RA-008-C8RA03569E-s001 [file RA-008-C8RA03569E-s001.pdf]

Electronic Supplementary Material (ESI) for RSC Advances.
This journal is © The Royal Society of Chemistry 2018

Supplementary information

Molecular-scale structures of surface and hydration of bioinert mixed-charged self-assembled monolayers investigated by frequency modulation atomic force microscopy

Yuki Araki, Taito Sekine, Ryongsok Chang, Tomohiro Hayashi and Hiroshi Onishi

Table of contents

S1. Evaluation of packing density of SA and TMA molecules.

S2. Evaluation of ratio of SA and TMA.

S3. Confirmation of thermal drift in imaging.

S1. Evaluation of packing density of SA and TMA molecules

We evaluated the packing densities of the molecules from Au_{4f} signals that are attenuated by the molecules constituting the SAMs.

First, we made a reference plot of XP intensity as a function of the number of the electrons in the molecule using the SAMs of n-alkanethiols with different carbon atoms. As these molecules are packed into root 3 x root 3 R30 structure, the XP intensity decreases exponentially as a function of electrons (and number of carbon).

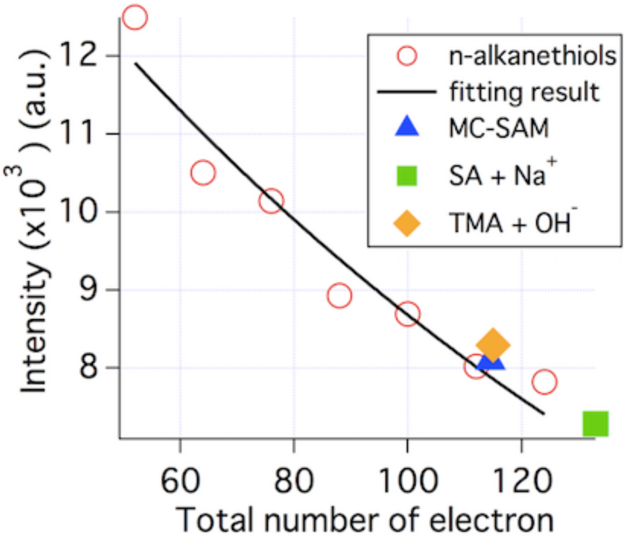

Fig. S1 XP intensity as a function of number of electron in the molecules constituting the SAMs. The take-off angle of the photoemission is 52 degree from the surface normal.

Then, we assume the "effective" number of electrons with assuming same packing structure as n-alkanethiols, and the packing densities were evaluated by dividing the effective numbers of electrons with the "real" total number of electrons.

T. Sekine, S. Asatyas, C. Sato, S. Morita, M. Tanaka and T. Hayashi, *J Biomater Sci Polym Ed*, 2017, **28**, 1231-1243.

T. Hayashi, Y. Tanaka, Y. Koide, M. Tanaka and M. Hara, *Phys. Chem. Chem. Phys.*, 2012, **14**, 10196-10206.

The packing density evaluated by XPS (90 ± 3%) with respect to the density of n-alkanethiols) is in good agreement with close to obtained by FM-AFM imaging (87%). We anticipate that the discrepancy originates in the defects in the SAMs, since the domain size of MC-SAM is relatively smaller than that of n-alkanethiols.

S2. Evaluation of ratio of SA and TMA

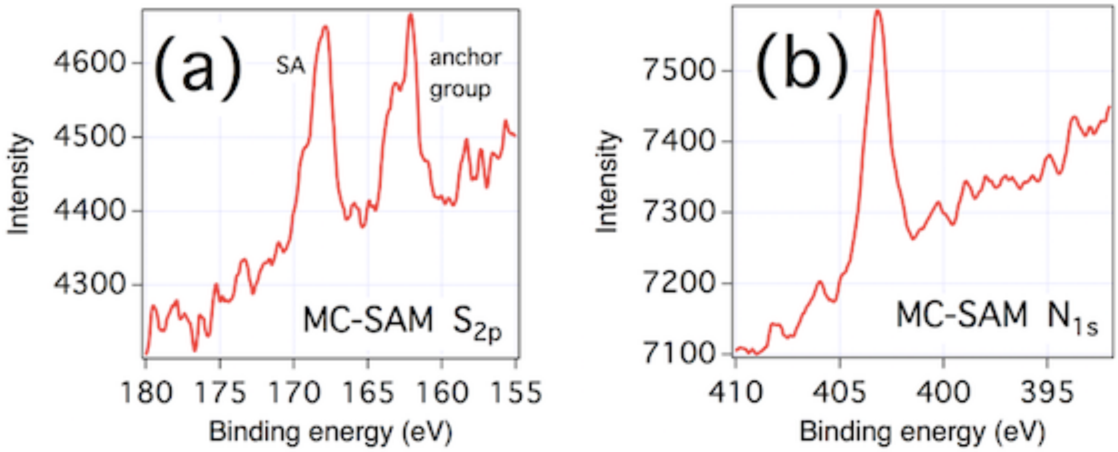

Fig. S2 XP spectra of MC-SAM for (a) S_{2p} and (b) N_{1s} states.

For the evaluation of the ratio between densities of TMA and SA molecules, we fabricated the pure SA and TMA SAMs and evaluated the packing densities of the molecules. We also measured S_{2p} and N_{1s} spectra and evaluated the packing density of SA and TMA molecules. Through this analysis, we confirmed that the ratio between the numbers of SA and TMA in MC-SAM is almost equal, i.e., the charge neutrality is maintained.

S3. Confirmation of thermal drift in imaging.

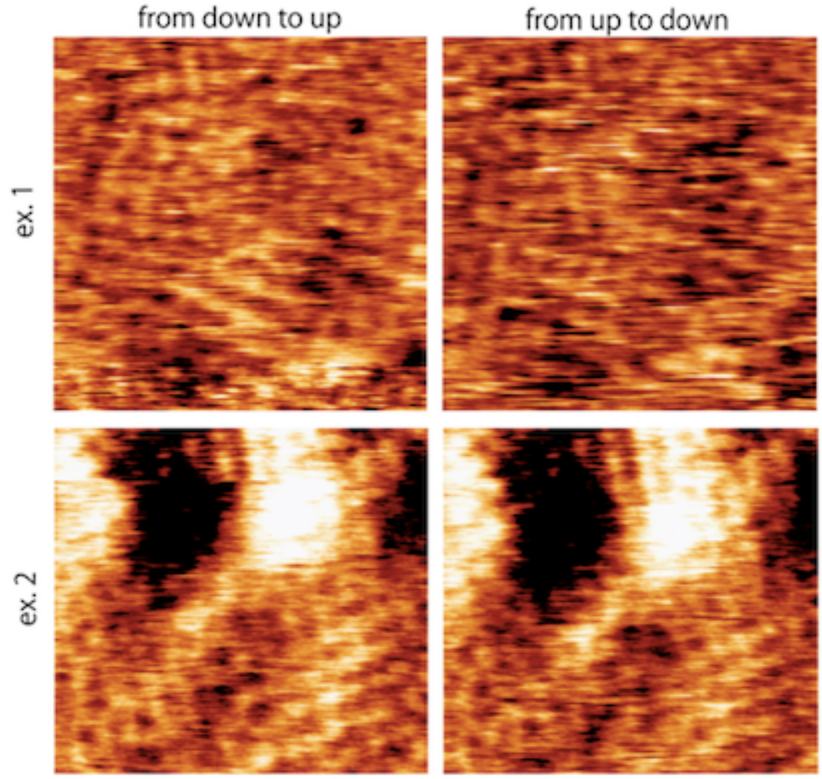

Fig. S3 Topographic images of MC-SAM surfaces of the up-and-down scans. Ex. 1 and 2 were observed at different scan areas. Scan area of all images was 5 nm.
